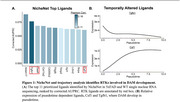# Pseudotime Analysis Identifies Receptor Tyrosine Kinase Ligands involved in the Temporal Development of Alzheimer's Disease Associated Microglia

**DOI:** 10.1002/alz70855_107399

**Published:** 2025-12-24

**Authors:** Brendan R Tobin, Sara Bitarafan, Levi B Wood

**Affiliations:** ^1^ Georgia Institute of Technology, Atlanta, GA, USA

## Abstract

**Background:**

In Alzheimer's Disease (AD) microglia transition their phenotype from homeostatic to a potentially destructive disease associated phenotype (DAM). Prior research identified the ERK1/2 protein as being hyperactivated in microglia isolated from an AD mouse model. ERK1/2 is activated by the binding of upstream receptor tyrosine receptors (RTK) with their corresponding ligand(s). Further, DAM development is a progressive process, where cells transition from a homeostatic to DAM phenotype over time.

**Method:**

A single nucleus RNA sequencing dataset from a 5xFAD mouse model was obtained from the Gene Expression Omnibus database (GSE140511). 3 wildtype (WT) and 3 5xFAD mice samples were used for this work. The transcriptomes from these samples were processed with Seurat (v.5.0.3). The microglial population of cells was identified and loaded into NicheNetR (v2.1.0) to identify ligands suspected of driving the differential gene expression in the 5xFAD microglia. Next, a single nuclear trajectory was established using Monocle3 (v1.3.7), where the homeostatic microglia were labeled as the pseudotime origin.

**Result:**

Several RTK ligands were identified by NicheNet as having high likelihood of driving the DAM phenotype gene expression (Figure 1A). They include *Csf1, Tgfb1*, and *Gas6*, which are largely expressed by microglia. Notably, each of these ligands have been established as altering microglia behavior outside of AD.

The Monocle3 trajectory of DAM development was then used to identify the stage in DAM development that the ligand may regulate. *Csf1* and *Tgfb1* changed as a function of pseudotime, shown in Figure 1B. *Csf1* signaling began early in DAM development and plateaued later. Alternatively, *Tgfb1* expression declined early in DAM development and further declined in the later stage. These trends align with what is known about each ligand in microglia, where Csf1 is proinflammatory and Tgfb1 is anti‐inflammatory. *Gas6* was generally expressed at the same level over time. This ligand is still suspected of regulating DAM, just not at a specific point of development.

**Conclusion:**

This analysis has identified three ligands for RTKs that are potential drivers of DAM and change expression temporally. Given their apparent role in DAM development, these ligands have therapeutic potential in AD through DAM modulation.